# HIV and cancer in Africa: mutual collaboration between HIV and cancer programs may provide timely research and public health data

**DOI:** 10.1186/1750-9378-6-16

**Published:** 2011-10-17

**Authors:** Sam M Mbulaiteye, Kishor Bhatia, Clement Adebamowo, Annie J Sasco

**Affiliations:** 1 Infections and Immunoepidemiology Branch, Division of Cancer Epidemiology and Genetics, National Cancer Institute, NIH, DHHS, Rockville, MD 20852, USA; 2 Institute of Human Virology, School of Medicine, University of Maryland, Baltimore, MD 21201, USA; 3 Epidemiology for Cancer Prevention, Team of HIV, Cancer and Global Health in Resource Limited Countries, Inserm U 897, Bordeaux Segalen University, Bordeaux, France

## Abstract

The eruption of Kaposi sarcoma (KS) and aggressive non-Hodgkin lymphoma (NHL) in young homosexual men in 1981 in the West heralded the onset of the human immunodeficiency virus (HIV) infection epidemic, which remains one of the biggest challenges to global public health and science ever. Because KS and NHL were increased >10,000 and 50-600 times, respectively, with HIV, they were designated AIDS defining cancers (ADC). Cervical cancer (CC), increased 5-10 times was also designated as an ADC. A few other cancers are elevated with HIV, including Hodgkin lymphoma (10 times), anal cancer (15-30 times), and lung cancer (4 times) are designated as non-AIDS defining cancers (NADCs). Since 1996 when combination antiretroviral therapy (cART) became widely available in the West, dramatic decreases in HIV mortality have been observed and substantial decrease in the incidence of ADCs. Coincidentally, the burden of NADCs has increased as people with HIV age with chronic HIV infection. The impact of HIV infection on cancer in sub-Saharan Africa, where two thirds of the epidemic is concentrated, remains poorly understood. The few studies conducted indicate that risks for ADCs are also increased, but quantitatively less so than in the West. The risks for many cancers with established viral associations, including liver and nasopharynx, which are found in Africa, do not appear to be increased. These data are limited because of competing mortality, and cancer is under diagnosed, pathological confirmation is rare, and cancer registration not widely practiced. The expansion of access to life-extending cART in sub-Saharan Africa, through programs such as the Global Fund for AIDS, Malaria, and Tuberculosis and the US President's Emergency Program for AIDS Relief (PEPFAR), is leading to dramatic lengthening of life of HIV patients, which will likely influence the spectrum and burden of cancer in patients with HIV. In this paper, we review current literature and explore merits for integrating cancer research in established HIV programs to obtain timely data about the incidence and burden of cancer in HIV-infected persons in Africa.

## Introduction

The global cancer burden has been increasing rapidly over the past 30 years [1], both in developed and developing countries [2]. The number of cases almost doubled from 7.6 million in 2002 to 12.7 million in 2008 [3], and are projected to continue increasing at 70% per year over the next 20 years [4]. The emergence and alarming spread, of the human immunodeficiency virus (HIV) epidemic has contributed to these increases. Heralded by eruption of Kaposi sarcoma (KS) [5,6] and aggressive non-Hodgkin lymphoma (NHL), including Burkitt lymphoma (BL) [7,8] in homosexual men in New York in 1981, the HIV epidemic has impacted the burden and trend of cancer in different countries. Abrupt increases in the number of KS cases in countries in sub-Saharan Africa, where KS was endemic [9,10], and in different countries in Europe [11], where KS was rare, signaled the pandemic nature of HIV and the general impact of the epidemic on cancer [12-14]. KS and aggressive NHLs, because of their dramatically elevated risk (100,000 and 282, respectively, in the U.S. [15]) with HIV and cervical cancer (CC) with less dramatic increase (10 times), were categorized as AIDS defining cancers (ADCs) to facilitate AIDS surveillance. Only a few other cancers were noted to be modestly increased with HIV and were categorized as non-AIDS-defining cancers (NADCs) [16]. While ADCs contributed the majority of cancer early in the AIDS epidemic, NADCs have assumed greater importance as survival has lengthened and patients are aging with HIV. Cancer now is estimated to contribute up to one third of deaths in patients with HIV in developed countries [17,18].

Although 70% of the global HIV/AIDS epidemic is concentrated in sub-Saharan Africa [19,20], the impact of HIV on cancer in this region is incompletely described. The impact of HIV on ADCs in Africa is similar, but less strong, than in the West [21,22]. Sparse data preclude detailed comparisons of pattern of NADCs, but dramatic increase in squamous cell carcinoma of the conjunctiva (SCCC) in many countries in Africa suggest the patterns differ from those observed in the West [21,22]. Behavioral and environmental risk factors account for the bulk of cancer in developed countries [23]. Conversely, infections account for proportionately more cancers in sub-Saharan Africa [24]. Thus, the impact of HIV on cancer in Africa might be expected to be different. In this paper, we summarize the impact of HIV on selected cancers in Africa, based on a panel discussion at the 12^th ^International Conference of the Institute of Human Virology at Tropea, Italy, in 2010. We summarize the consensus that collaboration with infectious disease HIV programs in sub-Saharan Africa may provide practical opportunities for research, treatment and prevention about cancer in HIV infected populations.

### HIV and cancer in the West

The bulk of our knowledge about HIV and cancer comes from studies conducted in the West (2448 of 2587 case-referent studies [21]), although this region is home to about 8% of the HIV epidemic [19,22]. KS, high-grade B-cell NHL, and cervical cancer (CC) were classified as ADCs based on data from this region [20,25], and the studies have shown that risk of KS and NHL, but not CC, increase with level and duration of immunosuppression [15,26,27].

KS was rare in the United States before the AIDS epidemic. Among white men in San Francisco, an early epicenter of the AIDS epidemic, incidence of KS rose steeply from 0.5 per 100,000 people/year in 1973 to a peak of 33.3 in 1991 [28], but mirroring trends in HIV/AIDS, the incidence fell to 2.8 in 1998. The risk for KS among persons with AIDS in the United States as compared with the general population was 22,100 during 1990-95 and 3,640 during 1996-2002 [29]. HIV/AIDS-related KS was estimated to account for about 81.6% of all KS cases in the U.S. during 1980-2007 [30]. The contribution of AIDS-related KS as a percentage of total KS burden in the United States peaked at 90.5% during 1990-95 and declined to 70.5% during 2001-07 [30]. These changes are related to use of combination anti-retroviral therapy (cART).

In contrast to KS, NHL was relatively common in the general population in the U.S. and the incidence was rising before the arrival of the HIV epidemic [28]. Impressive increases were noted for aggressive NHLs, including diffuse large B-cell lymphoma (DLBCL), BL, and CNS lymphoma[31]. For example, NHL rates among white men in San Francisco rose from 10.7 in 1973 to peak at 31.4 in 1995 then declined to 21.6 in 1998, but the incidence rates increased more steeply for DLBCL, BL, and CNS lymphoma [28]. The risk for NHL among persons with AIDS in the United States as compared with the general population was 53.2 during 1990-95 and 22.6 during 1996-2002 [29]. The lifetime cumulative risk of NHL was about 10% [32,33]. The proportional contribution of AIDS-related NHL subtypes to all NHL peaked in the early 1990s (10.2% for DLBCL, 27.8% for BL, and 48.3% for CNS lymphoma) then declined to 4.7%, 21.5%, and 12.9% for DLBCL, BL, respectively, during 2001-07 [30].

The risk pattern for CC contrasts that of KS and NHL. Modest risk elevation of 4.2 times during 1990-95 and 5.3 during 1996-2002 was noted [29]. The proportional contribution of AIDS-related CC in the United States during 1980-2007 was low at only 0.4% of all cases [30], although it has increased from 0.1% during 1980-89 to 0.71% during 2001-07 [30]. In contrast to KS and NHL, CC is not associated with level and duration of immunosuppression [15,26,27]. Possibly, screening for CC has capped its incidence in the West.

The introduction of cART in the West in 1996 [34,35] and its fast scale-up resulted in rapid and sustained reductions in mortality from AIDS and in the incidence of KS and NHL, but not CC [16,36,37]. The substantial reduction in risk for KS and aggressive NHLs following widespread introduction of cART is consistent with the hypothesis that HIV influences risk for cancers via cellular immunosuppression and impairment of oncovirus immunosurveillance [16].

The incidences of several other cancers, including lung, anus, liver, and Hodgkin lymphoma are increased with HIV/AIDS. These cancers are currently considered NADCs, and the reasons for the increased incidences are varied. For example, lung cancer incidence has consistently been shown to be increased 3-4 times higher in persons with HIV/AIDS [30,38,39]. A high prevalence of cigarette smoking in persons with HIV/AIDS compared to the general population is generally believed to explain this increase, although, other cofactors, including altered pathophysiology of the lung with HIV infection, may contribute [40]. For anal cancer, co-infection with HPV is thought to be a key factor[41], while uncontrolled EBV infection might contribute to Hodgkin lymphoma increase[26].

Intriguingly, breast and prostate cancer incidences rates appear decreased with immunosuppression [15,42]. The reasons for this pattern are not well understood, but they may include direct and/or indirect effects of HIV infection on breast cells [43]. For breast cancer, Hessol and colleagues [44] postulated that CXCR4-expresing HIV virions reduce breast cancer risk by inducing apoptosis of neoplastic breast cells via interaction with the CXCR4 receptor, which is expressed on some breast cancer cells. Conversely, the prostate cancer deficit in people with HIV/AIDs seems to be related to early case detection in this group because greater access to prostate cancer screening using prostate specific antigen [42].

The incidence rates of most common epithelial cancers, such as colon cancer, are not increased [15,38]. Absence of a generalized cancer epidemic in the setting of HIV has cast doubt on the hypothesis that immunological surveillance for tumor plays a major role in keeping progression to cancer in check. However, long-term effects of HIV on cancer will become clearer as we study the spectrum of NADCs in aging HIV infected population on long-term treatment with cART [39,45].

### HIV and cancer in sub-Saharan Africa

Although the region is home to about 70% of the AIDS pandemic, only about 139 (5%) studies have been conducted to examine the impact of HIV and cancer in sub-Saharan Africa [21]. One of these studies used HIV/AIDS-cancer record linkage methods, in Uganda [46], which demonstrated the feasibility of using this approach to study cancer in poor countries. About 681,000 people are diagnosed with cancer in sub-Saharan Africa annually [4]. Given the size of the HIV epidemic in the region, even a small impact of HIV on cancer can result in substantial increase in cancer burden, especially as longevity increases due to widespread access to cART. KS and squamous cell carcinoma of the conjunctiva (SCCC) exhibit the strongest quantitative association with HIV/AIDS in sub-Saharan Africa [47-50]. The impact of HIV on cancers with established virus-associations, including BL, liver cancer, and nasopharyngeal carcinoma, is less clear [51]. The impact of HIV on ADC is summarized in Table 1 and reviewed below.

**Table 1:** Association between AIDS-defining cancers with HIV in children and adults in studies conducted in sub-Saharan Africa

Cancer	Country	Subjects	OR (95%CI)	Reference
Kaposi sarcoma	Uganda	Children	94.9 (28.5-315)	Newton et al., 2001[47]
	Malawi	Children	93.5 (26.9-324)	Newton et al., 1995[67]
	Uganda	Adults	6.4 (4.8-8.4)	Mbulaiteye et al., 2006 ± [46]
	Rwanda	Adults	35 (8.2-207)	Newton et al., 1995[72]
	South Africa	Adults	22 (12.5-39)	Sitas et al., 2000[73]
	South Africa	Adults	47.1 (31.9-69.8)	Stein et al., 2000[74]
				
Non-Hodgkin lymphoma				
Burkitt lymphoma	Uganda	Children	7.5 (2.8-20.1)	Newton et al., 2001[47]
	Uganda	Children	2.2 (0.9-5.1)	Parkin et al., 2000[51]
	Malawi	Children	12.4 (1.3-116)	Mutalima et al., 2008[68]
	Malawi	Children	2.2 (0.8-6.4)	Mutalima et al., 2010[67]
	South Africa	Children	46.2 (16.4-130)	Stefan et al., 2011[69]
Non-Burkitt NHL	Malawi	Children	4.4 (1.1-17.9)	Mutalima et al., 2010[67]
	South Africa	Children	5.0 (0.9-27.0)	Stefan et al., 2011[69]
	Uganda	Adults	6.2 (1.9-20)	Newton et al., 2001[47]
	Uganda	Adults	6.7 (1.8-17)	Mbulaiteye et al., 2006 ± [46]
	South Africa	Adults	5.0 (2.7-9.5)	Sitas et al., 2000[73]
	South Africa	Adults	5.9 (4.3-8.1)	Stein et al., 2000[74]
				
Cervical cancer	Uganda	Adults	1.6 (0.7-3.6)	Newton et al., 2001[47]
	Uganda	Adults	2.4 (1.1-4.4)	Mbulaiteye et al., 2006 ± [46]
	South Africa	Adults	1.6 (1.1-2.3)	Sitas et al., 2000[73]
	South Africa	Adults	1.6 (1.3-2.0)	Stein et al., 2008[74]

± Results based on record-linkage study; estimates represent standardized incidence ratios comparing risk of cancer in persons with HIV to the general population where cancers arose. OR, odds ratio; 95%CI, 95% confidence interval.

### AIDS-defining cancers

#### Kaposi sarcoma

**Figure 1:**
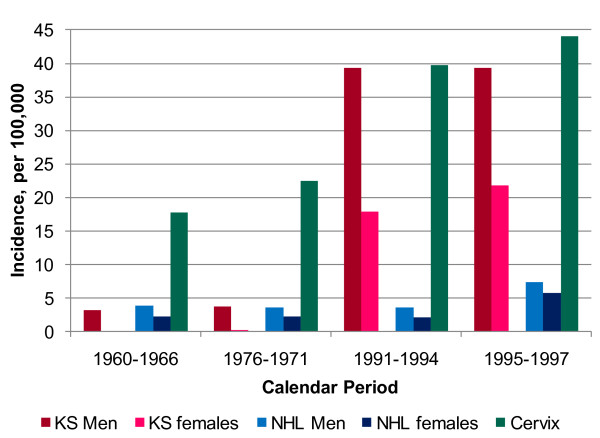
**Age-standardized incidence rates for AIDS-defining cancers (ADC) in Kyadondo County, Uganda for time-periods before and after the onset of the AIDS epidemic (early 1980s)**.

**Figure 2:**
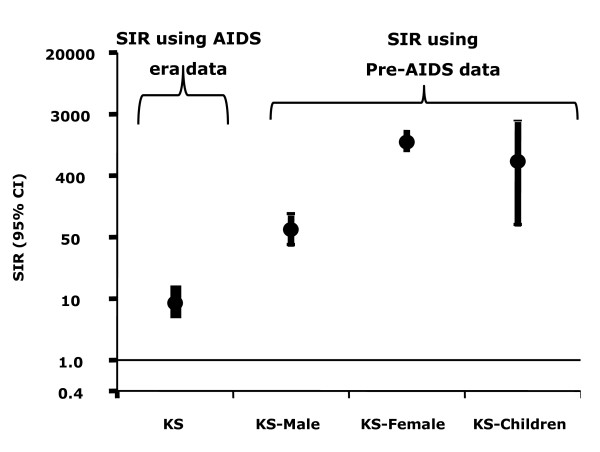
**Standardized incidence ratios for Kaposi sarcoma (KS) for all subjects based on KS rates calculated during the AIDS era (1989-2002) and pre-AIDS era (1961-1971) for men, women, and children**.

KS was endemic in East and Central Africa before the AIDS epidemic accounting for 5-18% of cancers [9,10]. During the AIDS epidemic, KS has become the most common cancer (Table 1) [52-54]. In Uganda, the annual age-standardized incidence rate per 100,000 men rose 12 times from 3.2 in 1960-66 to 39.3 in 1995-97 and from 0.1 to 21.8 per 100,000 women during the same periods (Figure 1) [52]. The proportion of KS in childhood cancers increased from 2% in the 1960s to 33% in the 1990s. The risk for KS with HIV/AIDS was elevated 6 times relative to KS rates in the contemporaneous general population in the Uganda HIV/AIDS Cancer match Study (Figure 2, SIR using contemporaneous, AIDS era, comparison data) [46]. The relatively modest increase in KS incidence in Africa compared to that observed in the West may be due to high background rates of KS and of HIV in the general population. In support for this explanation, the risk of KS in men, women, and children was much higher when the general population in the pre-AIDS KS years of 1960-1971 were used (Figure 2, SIR using pre-AIDS KS population incidence rates) as comparator. Another notable impact of HIV on KS was the loss of the large gender disparity in male/female incidence ratio from 20:1 in endemic KS to 2:1 in AIDS-related KS. The KS gender disparity (observed in classical KS as well) is not explained by differences in KSHV seroprevalence in men and women, which differ only by 20-50% [55]. Loss of gender disparity in the setting of immunosuppression suggests that immunocompetent women may be protected by gender-specific immunobiological factors [56], but the nature of these factors is unknown. A hypothesis that women might be protected by female hormones was advanced, but it not supported by reports that KS occurs in pregnant women[9,57] and laboratory studies have failed to find sex-hormone receptors on KS tissues [58]. KS risk is linked to environmental factors [59,60], KSHV viremia [61,62], expression of HIV proteins [63], immune reconstitution syndrome [64] and severe immunosuppression, some of which may contribute to gender disparity. Recently, Ruocco *et al.*, [65] has advanced the quinine or "oncodrug" hypothesis for KS in Africa, but this remains to be tested, and it would not explain the gender disparity. AIDS-related KS is a unique model of the relationship between viral infection, immunity, environmental, and genetic factors in viral cancers. Studies of KS patterns during the rollout of cART programs to document changes in the incidence and male/female ratio and the association, if any, with antimalarial use, may provide valuable insights into the biology of KS in Africa.

#### Non-Hodgkin lymphoma

In contrast to KS, the risk for NHL in the general population in sub-Saharan Africa is relatively low[66], except for childhood BL, which is endemic in some countries where it may accounts up to 50-75% of childhood cancers. Whether HIV has increased the risk of NHL risk in sub-Saharan Africa [67-69], especially of BL in particular, is unclear. The cumulative lifetime risk of NHL for AIDS patients in sub-Saharan Africa is about 3%, which is lower than about 10% observed in more developed countries at the onset of the epidemic [32]. In his autopsy study of 247 adult (>14 years) patients dying from AIDS-related conditions during 1991-1992, Lucas *et al.*, [70] found NHL in only 2.8% of HIV-positive decedents in Côte d'Ivoire. He estimated that the crude incidence of NHL was 84/100,000 per year among HIV-positive adults, about 10 times greater than the expected pre-AIDS incidence of NHL. None of 78 autopsied HIV-positive children (median age = 17 months) had NHL. Case series data of 26 adults (aged >16 years) with BL in Kenya during 1992-96 were compatible with an increase three times that expected from previous estimates [71]. The median age of HIV-positive patients with BL was 35 years compared with 16-25 years for those who were HIV-negative, and the HIV positive cases presented with lymph node involvement, indicating a departure, most likely as a result of the HIV epidemic, from the presentation typically seen in endemic BL. Recent reports from the Kampala Cancer Registry in Uganda have noted that NHL incidence rates increased three times from 2.3 per 100,000 persons in 1961 to 6.6 in 1997 (Figure 1) [52]. This increase was mostly due to pediatric BL (from 0.9 to 3.8 over the time period) and to DLBCL in young adults, which is compatible with an increase due to AIDS, but less so than observed among HIV-positive adults in industrialised countries.

The risk of NHL with HIV has been quantified in a few studies (Table 1). In Rwanda, Newton *et al. *[72] found an odds ratio (OR) for HIV of 12.6 (95%CI, 2.2-54.4), based on 19 clinically and histologically diagnosed NHL cases. In South Africa, Sitas *et al*. [73] found an OR for HIV of 5.0 (95%CI, 2.7-9.5), based on 105 histologically confirmed adult NHL cases compared with 844 hospitalized control subjects who had cancers unrelated to HIV (in adult men) or vascular disease (in adult women), enrolled at a tertiary hospital. These results have been confirmed in more recent analyses including larger numbers from South Africa [74]. The SIR for NHL was elevated 6.7 (95%CI, 1.8-17) times than in the general population in the Uganda HIV/AIDS-cancer match study [46]. However, the finding of marginal or null association with HIV has been reported at least one study in Uganda: OR of 2.2 (95%CI, 0.9-5.1), based on 38 histologically confirmed cases [51].

The reasons for the comparatively lower risk of NHL in sub-Saharan Africa relative to rates observed in the West may be artifactual due to under-diagnosis or may be due to competing mortality or environmental/genetic factors. Survival with HIV may be too short due to competing mortality from common infections, such as malaria and tuberculosis [75], to permit the significant development of NHL. This reason was supported by findings of fewer subjects with severely depressed CD4^+ ^counts in sub-Saharan Africa than in the West [76]. However, report of a median survival of 10 years from HIV sero-conversion in a longitudinal data from a rural cohort in southwest Uganda [77] is comparable to survival observed in western countries before effective antiretroviral drugs were introduced and the individuals have had the opportunity to develop NHL. Under-diagnosis, especially the limited availability of pathological diagnosis, may contribute [78]. Might racial factors contribute? Generally, NHL incidence is lower in African-American men and women than in white men and women [28], perhaps because of differences in environment, health care access and/or genetic factors. Possibly, the lower NHL risk with HIV in sub-Saharan Africa may reflect part of a general pattern of lower NHL risk in Blacks.

Is it possible that decreased risk of NHL might be due to prevalent exposure linked to treatment of common infections in the region? For example, antimalarials, including chloroquine, are widely used in Africa through formal prescription as well as self-medication for fever is prevalent [79]. In this regard, we are intrigued by recent animal data showing that administration of antimalarials to mice transgenic for c-myc inhibits B cell lymphomagenesis [80]. We are also intrigued by the widely accepted view that immune activation may contribute to HIV/AIDS-related lymphomagenesis [81]. If so, we would expect a high incidence of NHL in sub-Saharan Africa because immune activating infections, such as malaria, tuberculosis, are common in Africa. We observe the opposite. Is it possible that treatments for these conditions, by reducing immune activation, may coincidentally lower the risk of NHL as well? This hypothesis is compatible with recently published findings of reduced HIV-induced immune activation in a Ugandan cohort receiving antitubercular therapy [82]. Clearly, firm conclusion on these issues must await new data on the impact of HIV on NHL that will emerge from new studies that are being conducted with improved diagnostic and data capture methods.

#### Cervical cancer

Cervical cancer is the most common cancer in women in most countries in sub-Saharan Africa [47,73], but the impact of HIV on invasive CC is unclear. Screening programs for CC in sub-Saharan Africa are not well developed, so we would not expect to be able to readily demonstrate HIV-related increase in CC incidence. No dramatic increase has been noted in registry-based studies and clinical reports do not suggest a dramatic increase in number of cases, or drastic change in mean age incidence or disease stage at diagnosis [83]. The associations between CC and HIV have been small or null. For example, in Uganda, Newton *et al*., [47] observed an HIV prevalence of 32% in 65 women with CC attending four large referral hospitals in Kampala versus 21% observed in 112 controls with non-HIV related cancers or noncancerous conditions recruited at the same hospitals (OR, 1.6; 95% CI, 0.7-3.6). Similarly marginal results have been reported in a study conducted in South Africa, where the prevalence of HIV among 1323 cases of CC was 12.6% versus 9.0% observed in a comparison group of women hospitalized with a mixture of non-HIV-related cancers or vascular disease (OR, 1.6; 95% CI, 1.1-2.3) [47,73]. The risk for CC was 2.4 times (95% CI, 1.1-4.4) in women with HIV/AIDS in Kampala compared with women in the general population in the same area in the Uganda HIV/AIDS cancer match [46]. These results are compatible with a modest elevation in CC risk with HIV, and the conclusion that the impact of immunosuppression on CC risk is likely small.

The prevalence of cervical intraepithelial neoplasia (CIN) or human papilloma virus (HPV) infection is elevated 2-6 times in HIV-positive women than in HIV-negative women in east [84,85], west [86,87], and southern Africa [88], although not all studies agree [89-91]. In their study of Nairobi prostitutes in 1992, Kreiss *et al*., [89] found cervical HPV DNA in 37% of HIV infected women versus 24% in HIV non-infected women (OR, 1.7, 95% CI 0.8-3.6). The OR for HPV DNA in women attending an antenatal clinic in Mwanza in Tanzania was 1.02 (95%CI, 0.6-1.6) [90]. The link between squamous intraepithelial lesions (SIL) and immunity has not been fullu characterized [92]. The risk of low grade SIL was 6.1 (95%, CI = 1.2-41.4) times elevated in women with CD4+ cell count < 200/mm^3 ^compared to those with higher counts [92], although these findings were based on 20 subjects with SIL. In a larger study including 710 HIV positive women in Rwanda, Anastos *et al., *[93], found cervical HPV DNA (HPV 16 contributed 14%) in 67% of the women, of whom 8.8% also had CIN grade 3. Detection of HPV other than 16 was inversely associated with CD4 counts. Interestingly, a positive association between malaria infection in the past 6 months and risk of CIN3, which has not been reported before, was observed. The risk of high grade SIL was 2.4 times higher in HIV positive women with <200 CD4/ml than HIV positive women with >500 CD4/ml in a study of 1,010 HIV positive women in South Africa [94]. In this study, detection of HPV 16 and 66 were inversely related to CD4 count. Taken together, the modest increase in CC with HIV is compatible with a small impact due to HIV, but the relationship with immunity is observed only with pre-invasive CC lesions.

Despite the small risk increases reported, CC is the most important cancer in HIV infected populations in Africa because women account for >50% of HIV epidemic. The public health impact of CC could be addressed by harnessing the historical interest and increased funding of HIV/AIDS treatment and prevention programs to support new initiatives for CC screening and treatment [95]. This approach has been attempted in Zambia with great success [95,96]. The Zambian model has brought 58,000 women, including HIV negative women, for CC screening up from 0 less than 5 years ago by leveraging available, broad-based capacity-building efforts of vertical HIV/AIDS care and treatment programs. These modest programs are saving about one CC death per 46 HIV-positive women screened, demonstrating potential public health benefit. The program will yield timely data on HPV infection and risk for cervical dysplasia among HIV-infected women in Africa [93], and might make it possible to examine novel hypotheses, such as the interaction between malaria and CC [93].

### Non-AIDS-defining cancers

#### Squamous cell carcinoma of the conjunctiva

Squamous cell carcinoma of the conjunctiva (SCCC) is a rare tumor of the ocular surface, which is linked to ultraviolet radiation exposure and, based on elevated risk with HIV [97], appeara to be etiologically linked to immunosuppression and/or an underlying, albeit not yet well-characterized infection. The link with HIV infection was first reported by Ateenyi-Agaba in 1995 [50] when he observed that 75% of patients with SCCC at Mulago Hospital in Uganda were HIV-seropositive compared with only 19% of cases with nonmalignant eye conditions. This finding was confirmed in other case-control studies conducted in the tropics and subtropics [97,98], but it has not been reported in South Africa [74], suggesting that ultraviolet exposure may be a necessary component of the HIV impact. The risk of SCCC in Uganda 15 times from before to during AIDS. The incidence rate of ocular tumors, which are mostly due to SCCC, in Kyadondo County rose from 0.2 per 100,000 person-years in 1960-66 to 3.0 in 1995-97. The proportion of SCCC in eye tumors during the same period increased from 23.5 to 71% in men and 0 to 85% in women [52]. The dramatic increase in SCCC focused attention on the role of immunity and infection in SCCC, prompting a search for infectious etiology, including of mucosal or genital high-risk and cutaneous HPV [99-103]. de Koning *et al. *have summarized the comprehensive literature on the association between HPV and SCCC [103] The studies conducted have reported a higher prevalence of cutaneous HPV, but not genital HPV types, in SCCC (OR ranges 8 - infinity) [103]. The frequency or load of HPV DNA does not appear to vary with histological grade of tumor [103]. The studies conducted thus far remain are relatively small, and the positive results may be biased, while differences in laboratory methodology for HPV detection is a limitation likely shared by all the studies. Nonetheless, the consistent finding of elevated risk of SCCC with HIV supports the hypothesis that a known or novel HPV or other infectious agents may be involved in SCCC etiology, while the absence of impact in South Africa, suggests effects of immunosuppression are expressed on a background of exposure to ultraviolet radiation.

#### Hodgkin lymphoma

Hodgkin lymphoma, although not designated as AIDS defining, is consistently elevated with HIV in most studies [15,104]. The risk was elevated in the Uganda HIV/AIDS cancer match study (OR, 5.7; 95%CI, 1.2-17) and in a case-control study conducted in South Africa (OR, 1.4; 95%CI, 1.0-2.7). Interestingly, some [105] but not all studies [106] conducted in the West have reported that risk for Hodgkin lymphoma with use of cART. Whether Hodgkin lymphoma risk will further increase as cART becomes widely available in sub-Saharan Africa will be clarified by follow-up studies of cohorts on cART in Africa.

#### Liver cancer

Hepatocellular carcinoma (HCC) was relatively common in men in Africa before the AIDS epidemic, in part, because of the high prevalence of hepatitis B virus (HBV) infection and exposure to aflatoxin[66]. There is no evidence that HCC risk has increased during the AIDS epidemic, although data for deep seated tumors in Africa should be considered largely incomplete. The rates of liver cancer in Uganda, one of the first countries to be touched by the AIDS epidemic[107], were stable among men, but they increased by 50% among women from 1960-80 to 1991-05 [108]. Given the high prevalence of chronic HBV infection, aflatoxin exposure, and to a lesser extent hepatitis C infection in sub-Saharan Africa [109,110], a surge in HCC cases might have been expected. This reasoning is supported by recent data suggesting that HIV might be associated with significant liver fibrosis [111]. However, as liver cancer has a long induction period, the associations with HIV might not be expected early in the HIV/AIDS epidemic, and lower risk might be related to under-diagnosis, non-pathological confirmation, and short survival. Continued surveillance of HCC might provide improved understanding of the impact of HIV in HCC risk in sub-Saharan Africa.

#### HIV and cancer in sub-Saharan Africa: caveats and opportunities

Evaluation of cancer statistics must focus on the quality of data available to support scientific and public health initiatives. Although based on the best data currently available, the impact of HIV on the incidence and burden of cancer is probably underestimated. Only about one third of people with HIV in sub-Saharan Africa know about their infection [112] and only about one third of those who need HIV-specific treatments are receiving them [46,113]. In addition, cancer registration is relatively underdeveloped [114]. Although record-linkage methods, which have been used effectively and efficiently in the West, are feasible in Africa [46], lack of high-quality computerized medical records [115] precludes use of some other effective methods.

The study of cancer in HIV persons in Africa is valuable for several reasons. First, the large size of the HIV epidemic underscores its public health significance, including in its impact on cancer. Two, Africa encompasses extraordinary genetic diversity of pathogens and hosts, which hold promise for unique opportunities to learn about the biology of infection, immunology and cancer. Two HIV types are relevant to the epidemic (HIV-1 and HIV2), but HIV-1 is responsible for 95% of HIV infections globally. HIV-1 is divisible into ten subtypes (A-H, J, K) and some circulating recombinant forms are recognized [116], which have different transmission potentials and pathogenesis and could, plausibly, be associated with differential risk of cancer, and vary by geography. HIV-2 infection has a restricted distribution confined to West Africa, where co-distribution with HIV-1 offers opportunity to investigate HIV-type specific effects. For example, one small study including 40 women found that HIV-2 was associated with increased HPV clearance [117] and women infected with HIV-2 were less likely to develop high-grade SIL (HR, 0.3; 95%CI, 0.1-0.9) than those infected with HIV-1 [118]. This finding should be interpreted with caution because the small size of the study, and it was not statistically significant when CD4 counts were taken into account. HIV-1, subtype C accounts for about half (48%) of all global HIV-1 infections [116], and is the major subtype in southern Africa. Subtypes A and D are predominant in equatorial sub-Saharan Africa, where they account for 12% and 2%, respectively, of HIV-1 infections. Subtypes B and G account predominate in the West and the Far East Africa and they account for 11% and 5%, respectively, of HIV-1 infections. Inter-HIV-1 subtype recombinants are playing an increasingly important role. They account for 20% of infections occurring in all regions. There are rare subtypes such as F, H, J, and K, which together account for <1% of infections [116]. Most current knowledge about the link between HIV and cancer risk is based on populations with subtype B, highlighting our still very limited knowledge of interactions between HIV, host immunogenetics, and cancer.

Globally coordinated efforts aimed at interrupting the spread of, and mortality from, HIV/AIDS in sub-Saharan Africa present opportunities to study cancer in individuals with HIV/AIDS on the continent [19]. Millennium Development Goal (MDG) 6, established by the UN General Assembly Special Session in 2000 to halt and reverse the spread of HIV-1, malaria, and other diseases, led to the creation of the Global Fund for AIDS, Tuberculosis, and Malaria and unprecedented opportunities for funding. The US President's Emergency Plan for AIDS Relief (PEPFAR) launched shortly thereafter provided additional funding streams for HIV prevention and clinical care [119,120]. These initiatives, which largely ignore cancer [121], have created a funding stream that has seen access to HAART increase from <1% in 1999 to 35% in 2009 [19] and resulted in dramatic declines in the rate of new infections, stabilization of the epidemic, reduction of AIDS-related mortality, and increase in life expectancy [122]. This funding stream has strengthened infrastructure for HIV/AIDS disease surveillance, diagnosis, and data capture and established large or networked cohorts [115,123]. The resulting infrastructure and cohorts could be converted to provide new data on cancer in sub-Saharan Africa. For example, more than 13,000 PEPFAR clinics provide HIV prevention and treatment to millions of individuals in sub-Saharan Africa. Similarly, the International Epidemiology Databases to Evaluate AIDS (IeDEA), a global consortium funded by the National Institutes of Health, has linked 183 clinics in 17 countries in sub-Saharan Africa serving 286,793 individuals [124]. These large linked clinics offer three broad-based opportunities: to define: a) the spectrum of common and rare cancers in HIV-infected individuals and to obtain precise estimates of risk and heterogeneity of risk; b) temporal trends on common and rare cancers, including before and after introduction of cART; and c) test specific hypothesis, such as investigating the risk of KS with immune reconstitution syndrome [64,125], the role of antimalarials in NHL or other cancers, and the role of genetic and/or viral co-infections in cancer [20], or assess impact of interventions. We think the opportunities may be best considered along the lines of a resource-focused and disease-focused approach, as discussed below.

#### Resource-focused approach

The resource-focused approach would aim to leverage large networked or linked clinics [124,126,127] to routinely collect data that can be used for multiple studies and purposes. In the West, efforts such as the Swiss Cohort Studies [128] and the U.S. HIV/AIDS Cancer Match Study [29] provide useful models to consider. Because cancer is rare even in people with HIV, combining data from different cohorts allows investigators to address questions pertaining to HIV/AIDS that cannot be answered in small single institution cohorts. We are encouraged by establishments of such cohorts in Africa, prominently including the IeDEA consortium (http://www.iedea-hiv.org), which was launched with funding from the National Institutes of Health, to collect, harmonize, and standardize data from the continent to allow comparative analysis of common and dissimilar impacts [129]. The African Organization for Research and Training in Cancer (AORTIC) (http://www.aortic.org/) has a mission is to promote cancer awareness and improve cancer diagnosis and treatment in Africa. With its network across the continent and bi-ennial scientific meetings, AORTIC provides a resource that could be leveraged to initiate, implement, and report on Africa-wide studies of cancer. The recent focus large biomedical centers, such as the National Cancer Institute at NIH, by establishing Centers for Global Health[130] is timely and likely to speed up the conversion of infectious disease-specific programs in developing countries into programs for study and control of cancer.

#### Disease-focused approach

In contrast to the resource-focused approach, in the disease-focused approach, specific hypotheses are raised and specific studies designed to answer those questions. For example, the question of the types and risk of NHL is currently being addressed by a consortium of investigators focused on lymphomas (http://www.ssalc.org/acsr_drupal/). These investigators will bring clarity to the question of the spectrum of lymphomas diagnosed with HIV in Africa and their diagnostic support is likely to spur epidemiological and clinical studies of NHL. We noted outstanding questions about the etiology of SCCC [97,99]. Disease-specific hypothesis-driven studies to investigate the role of immunosuppression, ultraviolet exposure, pathogens in the pathogenesis of the disease will bring clarity to our understanding of SCCC. Other interesting questions that could benefit from a disease-focused approach include understanding the association between HIV and lung and breast cancer. The risk for lung cancer is increased with HIV in the West, but given the much lower prevalence and intensity of cigarette smoking in sub-Saharan Africa, studies conducted there might shed light on the biology of the disease among non- or low-intensity smokers. The risk of breast cancer is decreased with HIV in the West [43]. Hessol *et al. *and co workers [44] have linked low breast cancer risk with HIV to infection with CXCR4-using variants of HIV. They speculated that CXCR4-using variants might reduce breast cancer risk by binding to apoptosis receptors on hyperplastic and neoplastic breast duct cells. This hypothesis brings new perspectives to the question about the biology for breast cancer with potential for treatment or intervention using CXCR4-agonists. Data about breast cancer in sub-Saharan Africa are sparse with some reporting increased risk [131,132] and others no increase [46]. Thus, clarifying the epidemiology of breast cancer in sub-Saharan Africa where CXCR4-using variants are prevalent may help evaluate the feasibility of testing and confirming or refuting this hypothesis. Finally, anal cancer is increased with HIV in the West [41]. Homosexual practices are being increasingly acknowledged in Africa, opening opportunity to investigate the association.

## Competing interests

The authors declare that they have no competing interests.

## Authors' contributions

SMM drafted the manuscript. All authors reviewed and critically revised the manuscript and approved the final draft.
